# 

**Published:** 1824-06

**Authors:** 


### MONTHLY CATALOGUE OF MEDICAL BOOKS.

*** *It having been hinted to us that gentlemen, sending copies of their Works, prefer having their titles given at length, it is our intention, in future, to comply with this suggestion: but it is to be observed, that no books can be entered on this List except those sent to us for the purpose;—finding, in the lists hitherto transmitted, that the names of works have frequently been given as published, which have not appeared for weeks, or even months after.*

Observations on Injuries of the Spine and of the Thigh-bone, in two Lectures delivered in the School of Great Windmill-street. The first in vindication of the Author's Opinions against the Remarks of Sir ASTLEY COOPER, Bart.; the second on the late Mr. JOHN BELL's Title to certain Doctrines, now advanced by the same Gentleman. Illustrated with nine Plates. By CHARLES BELL, Surgeon to the Middlesex Hospital.—4to. pp. 108. London, 1824.

An Elementary System of Physiology. By JOHN BOSTOCK, M.D. F.R.S. L.S. and H.S. M.R.I.; Member and V.P. of the Geological, Member and late V.P. of the Medical and Chirurgical, and Member of the Astronomical Societies of London; Member and late President of the Edinburgh Medical Society; Honorary Member of the Literary and Philosophical and of the Historical Societies of New York; Lecturer on Chemistry at Guy's Hospital; Professor of Physiology in the Liverpool Royal Institution, &c. &c. Vol. I.—pp. 503. London, 1824.

Surgical Anatomy of the Arteries of the Human Body, designed for the use of Students in the Dissecting Room. By ROBERT HARRISON, A.B. T.C.D. Member of the Royal Colleges of Surgeons in Ireland and London, and one of the Demonstrators of Anatomy in the School of Surgery in Dublin, under the Direction of the Court of Examiners of the Royal College of Surgeons. In two vols. Vol. I. —pp. 226. Dublin, 1824.

Recueil de Memoires, Consultations, et Rapports, sur divers Objets de Medecine Legale. Par M. F. CHAUSSIER, Chevalier de Saint Michel et de la Legion d'Honneur; Professeur a la Faculté de Medecine; Medecin en chef de la Maternité; Membre de l'Institut, et de l'Academie Royale de Medecine, &c.—pp. 519. Paris, 1824.

De la Moelle Epiniere et de ses Maladies, ouvrage couronné par la Societé Royal de Medecine de Marseille, dans sa seance publique du 23 Octobre, 1823. Par C. P. OLLIVIER D'ANGERS, Docteur de Medecine de la Faculté de Paris, Membre correspondant de la Societé de Medecine de Marseilles.—pp. 404. Paris, 1824.

Transactions of the Association of Fellows and Licentiates of the King's and Queen's College of Physicians in Ireland. Vol. IV.—pp. 452. Dublin, 1824.

On the Nature and Symptoms of Cataract, and on the Cure of that Disease in its early Stages, by a mode of Practice calculated to prevent the occurrence of Blindness, and to render unnecessary the Operations of Couching and Extraction. Illustrated by Cases. By JOHN STEVENSON, Esq. Fellow of the Royal College of Surgeons, and Surgeon-Oculist and Aurist to his Royal Highness the Duke of York, &c.—pp. 234. London, 1824.

Observations on Fever. By R. WADE, Member of the Royal Coll. of Surgeons, and Apothecary to the Westminster General Dispensary.—pp. 83. London, 1824.

Burgess and Hill's Quarterly Catalogue of Second-hand Books, in Anatomy, Medicine, Surgery, Midwifery, Chemistry, Veterinary Art, &c. &c. No. I. June 1824.—pp. 24. To be published every three months.

De l'Influence des Agens Physiques sur la Vie. Par W. F. EDWARDS, D.M. Membre Associé de l'Academie Royal de Medecine de Paris; Membre de la Societé Philomatique, de la Societé de Medecine de Dublin, &c.—pp. 654. Paris, 1824.

A Treatise on the Internal Use of the Natural and Factitious Waters of Carlsbad, Marrebad, Ems, &c. By Dr. F. KREYSIGE, of Dresden; Physician to his Majesty the King of Saxony; Knight of the Order of Merit and Fidelity; Honorary Member of the Medical and Chirurgical Society of London, &c. &c. Translated from the German, by GORDON THOMSON, M.D.—pp. 95. London, 1824.

### NOTICE TO CORRESPONDENTS.

*The Communications of* Dr. VANDEBURGH, Mr. ELLERT, Mr. JUKES, *and* Mr. MELIN, *were too late for insertion in the present Number.*

Mr. BUSH'S *Paper cannot be admitted* in its present form.

*The anonymous Communication "on the Virtues of the Succus foliorum Solani tuberosi" cannot be admitted.*

